# Transoceanic Spreading of Pathogenic Strains of *Vibrio parahaemolyticus* with Distinctive Genetic Signatures in the *recA* Gene

**DOI:** 10.1371/journal.pone.0117485

**Published:** 2015-02-13

**Authors:** Narjol González-Escalona, Ronnie G. Gavilan, Eric W. Brown, Jaime Martinez-Urtaza

**Affiliations:** 1 Center for Food and Applied Nutrition, Food and Drug Administration, College Park, MD, United States of America; 2 Centro Nacional de Salud Publica, Instituto Nacional de Salud, Lima, Peru; 3 Department of Biology and Biochemistry, University of Bath, Bath, United Kingdom; University of Illinois at Chicago, UNITED STATES

## Abstract

*Vibrio* *parahaemolyticus* is an important human pathogen whose transmission is associated with the consumption of contaminated seafood. Consistent multilocus sequence typing for *V. parahaemolyticus* has shown difficulties in the amplification of the *recA* gene by PCR associated with a lack of amplification or a larger PCR product than expected. In one strain (090–96, Peru, 1996), the produced PCR product was determined to be composed of two *recA* fragments derived from different *Vibrio* species. To better understand this phenomenon, we sequenced the whole genome of this strain. The hybrid *recA* gene was found to be the result of a fragmentation of the original lineage-specific *recA* gene resulting from a DNA insertion of approximately 30 kb in length. This insert had a G+C content of 38.8%, lower than that of the average G+C content of *V. parahaemolyticus* (45.2%), and contained 19 ORFs, including a complete *recA* gene. This new acquired *recA* gene deviated 24% in sequence from the original *recA* and was distantly related to *recA* genes from bacteria of the *Vibrionaceae* family. The reconstruction of the original *recA* gene (recA3) identified the precursor as belonging to ST189, a sequence type reported previously only in Asian countries. The identification of this singular genetic feature in strains from Asia reveals new evidence for genetic connectivity between *V. parahaemolyticus* populations at both sides of the Pacific Ocean that, in addition to the previously described pandemic clone, supports the existence of a recurrent transoceanic spreading of pathogenic *V. parahaemolyticus* with the corresponding potential risk of pandemic expansion.

## Introduction

*Vibrio parahaemolyticus* is a natural inhabitant of temperate and tropical coastal waters and the leading cause of seafood-borne gastroenteritis in the US, typically associated with eating raw or undercooked seafood [1]. Pathogenic strains of *V*. *parahaemolyticus* represent a small fraction of all environmental strains [2], and frequently carry genes encoding for thermostable direct hemolysin (*tdh*) and/or thermostable direct hemolysin-related hemolysin (*trh*) [3].

Until 1996, infections with *V*. *parahaemolyticus* were local phenomena, occurring in different areas of the world during warmer months of the year, and caused by region-specific strains. However, in 1996, over a period of months, infections with *V*. *parahaemolyticus* caused by strains belonging to a single clonal group, now referred as the “pandemic” clonal complex, were detected—initially in India and subsequently all across East Asia [4–9]. Infections associated with that pandemic clone were restricted to Asia, until their detection all along the Peruvian coasts and northern Chile throughout 1997, coinciding with the arrival and southwest geographical progression of the warm waters of El Niño [10]. This change in epidemiology was the first evidence that a pathogenic group of *V*. *parahaemolyticus* carrying the pandemic clonal complex could spread across such large distances. However, it has not yet been determined whether the transoceanic dispersal (by natural events such as the El Niño, or by human activities, such as transport of ballast water) of these pathogenic organisms was a singular event, or if long distance spreading is common events for the introduction of non-endemic variants of pathogenic vibrios in remote areas. The dominant mechanisms of transport and introduction could have significant impacts on public health in coastal communities around the world, and may influence the choice of preventative strategies.

Our knowledge of the population structure and diversity of *V*. *parahaemolyticus* populations has been greatly enhanced by advances in molecular typing techniques. Establishment of the multilocus sequence typing (MLST) scheme for *V*. *parahaemolyticus* [11] and centralized database (http://pubmlst.org/vparahaemolyticus) in 2008 has enabled researchers from around the world to compare isolates. Currently more than 1375 isolates from diverse regions of the world, belonging to 584 sequence types (STs), are available for analyses. Genetic variants prevailing in the different regions of the world can be mapped showing the links between populations from diverse geographical areas and drawing potential routes of dispersion. These MLST analyses reveal *V*. *parahaemolyticus* to be genetically diverse, with a weakly semi-clonal population structure [11], and emphasize the influence of environmental and oceanic dynamics on the demography of *Vibrio* populations [3,11–16]. The MLST database has enabled researchers to document the global distribution of the “pandemic” clonal complex [O3:K6 clone (CC3)] as well as two other major clonal complexes that prevail in different regions of the world [11].

Researchers have recently reported difficulties in amplifying one of the genes included in the MLST scheme: they have been unable to amplify the *recA* gene [17] or have obtained a PCR product almost double that expected for *recA* (*i*.*e*., PCR product of approximately 1400 bp rather than expected size of 729 bp [16]). This unexpected product was observed in one strain from Peru (090–96), isolated from clinical sources in 1996. Further investigation of this PCR product with Sanger sequencing showed it to be composed of two *recA* fragments from different vibrios. To provide a holistic understanding of this phenomenon, we sequenced the genome of strain 090–96. In addition, we compared the genome 090–96 to other previously sequenced *V*. *parahaemolyticus* genomes to assess whether strains with similar characteristics already exist in public databases, and to detect any potential epidemiological links.

## Materials and Methods

**Bacterial strain and media**. *V*. *parahaemolyticus* strain 090–96 strain was isolated from a stool sample in Chimbote, Peru, in March 1996 [18]. This *tdh+*/*trh-* isolate belonging to the O4:K8 serotype was retrieved from a -80°C freezer, transferred to Luria-Bertani (LB) medium with 3% NaCl and incubated at 250 rpm at 37 °C.

**DNA extraction and quantification**. Genomic DNA from each strain was isolated from overnight cultures using the DNeasy Blood & Tissue Kit (QIAGEN, Valencia, CA). The quality of the DNA was checked using a NanoDrop 1000 (Thermo Scientific, Rockford, IL) and the concentration was determined using a Qubit double-stranded DNA BR assay kit and a Qubit fluorometer (Life Technologies, Grand Island, NY) according to each manufacturer's instructions.

**Genome sequencing, assembly and annotation**. The isolate was sequenced using Ion Torrent (PGM) sequencing with the 200-bp read chemistry (Life Technologies, Carlsbad, CA) according to manufacturer’s instructions. The library was constructed using 2 μg of genomic DNA, previously sheared using a Covaris S220 System (Covaris, Inc., Woburn, MA) following a protocol to generate an average of 200 bp, and the SPRI works Library Preparation System III (Beckman Coulter, Indianapolis, IN). The resultant libraries were diluted to the recommended concentrations, determined by quantitative PCR (Life Technologies) according to the manufacturer’s instructions, to be used as templates for emulsion PCR (emPCR). The resultant enriched emPCR yield was loaded onto a 318 chip and sequenced using an Ion PGM 200 sequencing kit (Life Technologies) according to the manufacturer’s instructions. The initial analysis and identification of the strain was performed using an *in silico V*. *parahaemolyticus* MLST approach (CLC Genomics Workbench software version 6, CLC bio, Germantown, MD, USA) against a sequence of each of the seven alleles used in the MLST scheme and was found to be the same allele combination (dnaE11, gyrB48, dtdS48, pntA26, pyrC48, and tnaA26). Genomic sequence contigs were *de novo* assembled using default assembly settings within CLC Genomics Workbench with a minimum contig size threshold of 500 bp in length. These contigs were re-organized by Mauve analysis (http://gel.ahabs.wisc.edu/mauve), using *V*. *parahaemolyticus* strain RIMD2210633 as reference [19]. The draft genome was annotated using the NCBI Prokaryotic Genomes Automatic Annotation Pipeline (PGAAP, http://www.ncbi.nlm.nih.gov/genomes/static/Pipeline.html) [20].

**Whole genome phylogenetic analysis**. Forty-one public genomes for *V*. *parahaemolyticus* (Table A in S1 File) along with the 09–96 strain, comprising a dataset of 42 drafts and two complete genomes (RIMD2210633 and BB22OP), were analyzed. Phylogenetic analyses of these whole genomes were performed using the genome comparator available from the *V*. *parahaemolyticus* MLST website database (http://pubmlst.org/vparahaemolyticus). Briefly, the BIGSdb genome comparator tool performed a whole-genome MLST (wgMLST), producing a color-coded wgMLST output facilitating comparison among isolates. This output was used to categorize loci that were: 1) variable among all isolates, 2) identical among all isolates, 3) missing in all isolates, or, 4) incomplete due to being located at the ends of contigs. The set “variable loci among all isolates” was used for assessing their relationships, producing a distance matrix based on the number of variable alleles. The strains were then resolved into a network using the NeighborNet algorithm [21].

***in silico recA* region analysis and location**. In order to assess the location and organization of the *recA* region in strain 090–96, contig0035 (NCBI accession number: JFFP01000036) that contained the *recA* gene was annotated using two independent annotation services: BASys (https://www.basys.ca) and RAST server (http://rast.nmpdr.org) [22]. This was done because most of the genes in the DNA insertion region between the two *recA* fragments were annotated as hypothetical proteins using the NCBI pipeline. The annotation of contig0035 was then compared to the closest phylogenetic strain for which a genome is available (*i*.*e*., BB22OP; GenBank accession number: CP003973.1). Contig0035 was also compared to the contig containing the *recA* region from strain S130 (GenBank accession number: NZ_AWIW01000000), another strain in the NCBI database, whose genome contains a truncated *recA* with a similar insertion to that of strain 090–96. This later strain belongs to a different *V*. *parahaemolyticus* ST (ST527). A physical map of the *recA* gene region from these 4 strains was generated using easyFig 2.1 [23] with the nucleotide sequence comparison (BLASTn) of a fragment of contig0035 containing the insertion between the two *recA* fragments (original *recA* gene from strain 090–96) from positions 72530–106644.

**Assignment to clonal complexes**. The program eBURST v 3.0 was used to identify the different clonal complexes (http://eburst.mlst.net). The most restrictive group definition was used to define these clonal complexes, i.e., at least six of the seven alleles had to be identical to be included in the same group or clonal complex (CC) [24]. Statistical confidence of the ancestral types was assessed using 1000 bootstrap re-samplings. Two different STs are considered single locus variant (SLV) when they differ from each other at a single locus. Double locus variants (DLV) are any two different STs differing in two loci. An allele is any different variant in the sequence of a specific locus regardless of the number of single nucleotide polymorphisms (SNPs). If the sequence is different, it is considered to be a new allele and is assigned a unique (arbitrary) allele number.

**Phylogenetic analysis of the *recA* alleles**. The evolutionary history for the *recA* gene was inferred using the Minimum Evolution method (ME) [25], and corroborated using Maximum Likelihood method. A ME tree for *recA* locus from the *V*. *parahaemolyticus* MLST database was constructed by MEGA 6 software, [26] using the kimura-2 parameter method [27] to estimate the evolutionary distances and are in the units of the number of base substitutions per site. The optimal tree with the sum of branch length = 1.12753263 is shown. The tree is drawn to scale, with branch lengths in the same units as those of the evolutionary distances used to infer the phylogenetic tree. A neighbor-joining algorithm [28] was used to generate the initial tree. The statistical support of the nodes in the ME tree was assessed by 1000 bootstrap re-samplings. The analysis involved 242 nucleotide sequences. Codon positions included were 1st+2nd+3rd+Noncoding. All ambiguous positions were removed for each sequence pair. There were a total of 729 positions in the final dataset.

**Nucleotide sequences**. The draft genome of 090–96 strain (listed as CFSAN001595) was deposited in GenBank under the accession number JFFP00000000.

## Results and Discussion

**Draft genome assembly**. A draft genome was generated for *V*. *parahaemolyticu*s strain 090–96 with an estimated average coverage of 26X. Contigs were *de novo* assembled and the number of contigs, sequence lengths, and other assembly statistics for that strain are summarized in Table 1. The genome size and the average G+C content were approximately 5.1 Mb and 45.3% respectively, similar to that of other *V*. *parahaemolyticus* strains (genome sizes between 4.8 to 5.3 Mb, and average G+C content between 45.1 to 45. 6) in GenBank (Table A in S1 File). The assembled draft genome was annotated using the NCBI Prokaryotic Genomes Automatic Annotation Pipeline (PGAAP, http://www.ncbi.nlm.nih.gov/genomes/static/Pipeline.html) [20]. A total of 4,749 genes were identified, including 57 tRNAs. This number of genes is similar to that reported for other *V*. *parahaemolyticus* strains, which ranges from 4,591 (AQ4037) to 6,190 (VPCR-2010) (http://www.ncbi.nlm.nih.gov/genome/genomes/691).

**Table 1:** Summary report of the *de novo* assembly of 090–96.

	090–96
Size (bp)	5,069,445
GC content (%)	45.3
# Contigs	116
N50*	106,956
Minimum contig size (bp)	501
Maximum contig size (bp)	358,101
Average contig size (bp)	43,702

* The N50 statistic is calculated by summing the lengths of the biggest contigs until 50% of the total genome length has been reached. The smallest contig within this set is reported the N50 value of a *de novo* assembly.

**Fig 1:**
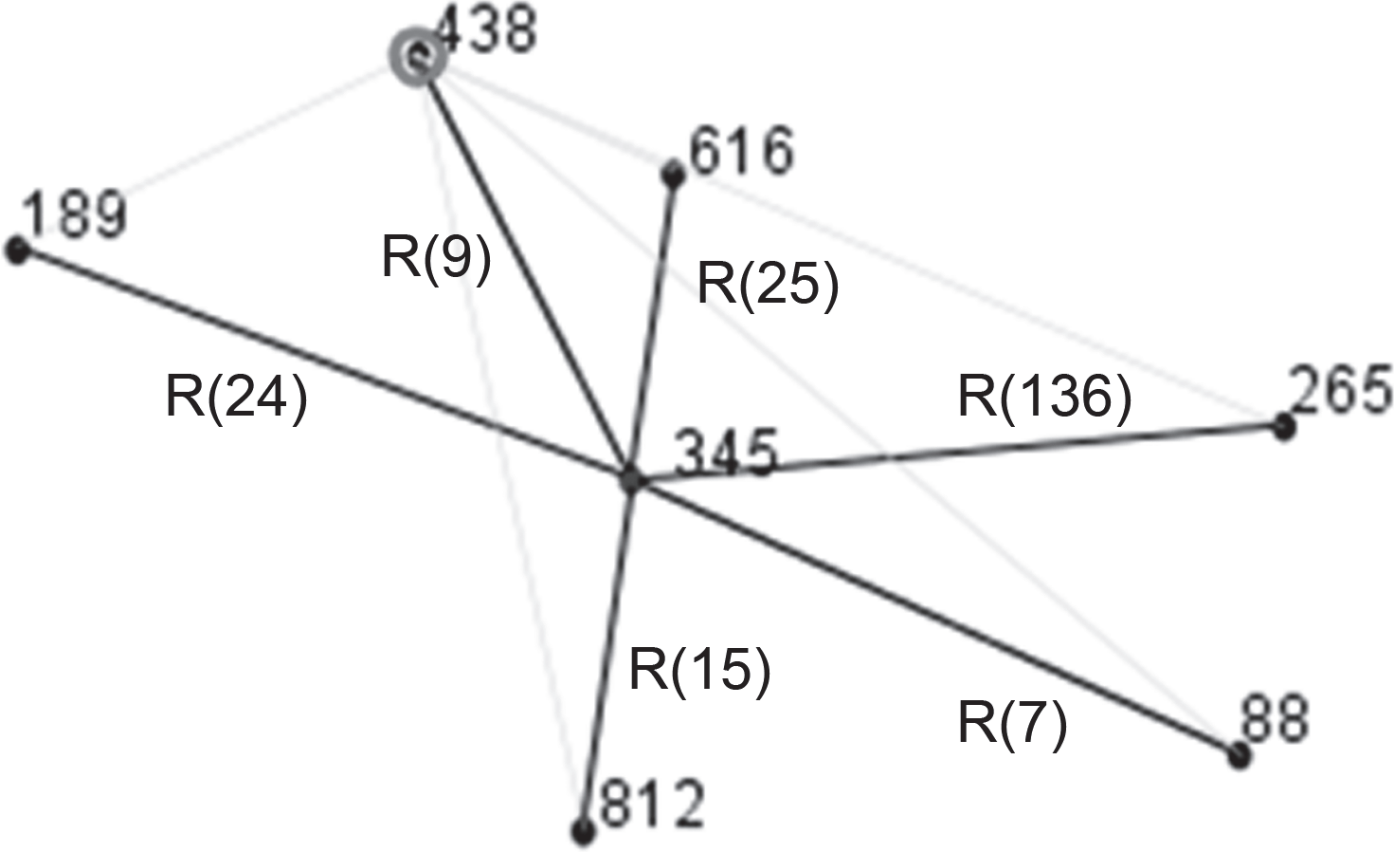
*V*. *parahaemolyticus* “snapshot” of CC345 obtained using eBURST v3. Other CCs were defined using stringent criteria (6/7 shared alleles) (Figure B in S1 File). Among those groups or CCs, ST345 was identified as the predicted clonal ancestor of CC345. STs that are SLV of each other are shown connected by lines (black). DLV STs are shown as connected light gray lines. CC—Clonal Complex. R- Recombination event and the number of SNPs between ST345 (founder) and the other STs are between brackets. All the differences were located at the *recA* allele, except for ST812 that were located at the *gyrB* allele.

***in silico* MLST**. Analysis of the *de novo* assembled contigs confirmed previous Sanger sequencing results for this strain by *in silico* MLST, available at http://pubmlst.org/vparahaemolyticus, with the following allelic profile: 11, 48, unknown, 48, 26, 48 and 26 for *dnaE*, *gyrB*, *recA*, *dtdS*, *pntA*, *pyrC*, and *tnaA* genes respectively. The allele type for the *recA* gene could not be assigned and was missing which impeded the assignation of the corresponding ST to this strain. Comparing 090–96 strain with strains already in the *V*. *parahaemolyticus* MLST database showed that the Peruvian strain was closely related to other strains, sharing from 5 to 6 allele types with them (Table 2). These strains were all part of a newly described clonal complex (CC) CC345 [29] (Fig. 1). ST345 was identified as the most probable founder of this CC. Diversity within this CC is driven primarily by recombination events (Fig. 1), which occur frequently at the *recA* gene, except ST812 in which changes were observed at the *gyrB* gene.

**Table 2:** *V*. *parahaemolyticus* strains with similar allele types to strain 090–96 available at the MLST *V*. *parahaemolyticus* database.

Strain	Isolation year	Country	Source	Serotype	gyrB	recA	ST
267–95	1996	Peru	C	O4:K8	48	43	88
324–95	1995	Peru	C	O4:K8	48	43	88
326–95	1995	Peru	C	O4:K8	48	43	88
NY477	1997	USA	C	unk	48	43	88
BB22OP	1982	Bangladesh	E	O4:K8	48	43	88
V445	2008	China	E	O4:K68	48	3	189
V254	2006	China	C	O3:KUT	48	3	189
VP200	1990	Thailand	C	O4:K8	48	3	189
S030_1379	1999	India	C	unk	48	3	189
S031_1441	1984	Japan	C	unk	48	3	189
VP013	2010	China	C	unk	48	19	345
VP11093	2011	China	C	O4:K8	108	19	438
VP190	1990	Thailand	C	O4:K10	48	107	265
VP07001	2007	China	E	O4:K8	48	107	265
VP08353	2008	China	C	O1:K56	48	82	812
VP08354	2008	China	C	O1:K56	48	82	812
SZ-VP08018	2008	China	C	O4:K8	48	112	962
VP08001	2008	China	C	O4:K8	48	unk	unk
VP11204	2011	China	C	O4:K8	48	unk	unk
VP11206	2011	China	C	O4:K8	48	unk	unk
090–96	1996	Peru	C	O4:K8	48	unk	unk
212–96	1996	Peru	C	O4:K8	48	unk	unk
232–06	2006	Peru	C	O4:K8	48	unk	unk
245–07	2007	Peru	C	O4:K8	48	unk	unk
691–05	2005	Peru	C	O4:K8	48	unk	unk
vp196	2002	Peru	E	O4:K8	48	unk	unk
1257–07	2007	Peru	C	O4:K8	48	unk	unk
1262–07	2007	Peru	C	O4:K8	48	unk	unk

In bold are Peruvian strains belonging to this CC345. VP08001, VP11204, VP11206 are Chinese strains with an identical profile and probably carrying the same insertion. Only loci showing different alleles among these strains are displayed in the table. The other loci were dnaE11, dtdS48, pntA26, pyrC48, and tnaA26 for all of them.

C- clinical. E- environmental. unk—unknown. UT- untypable. NY- New York.

**Fig 2:**
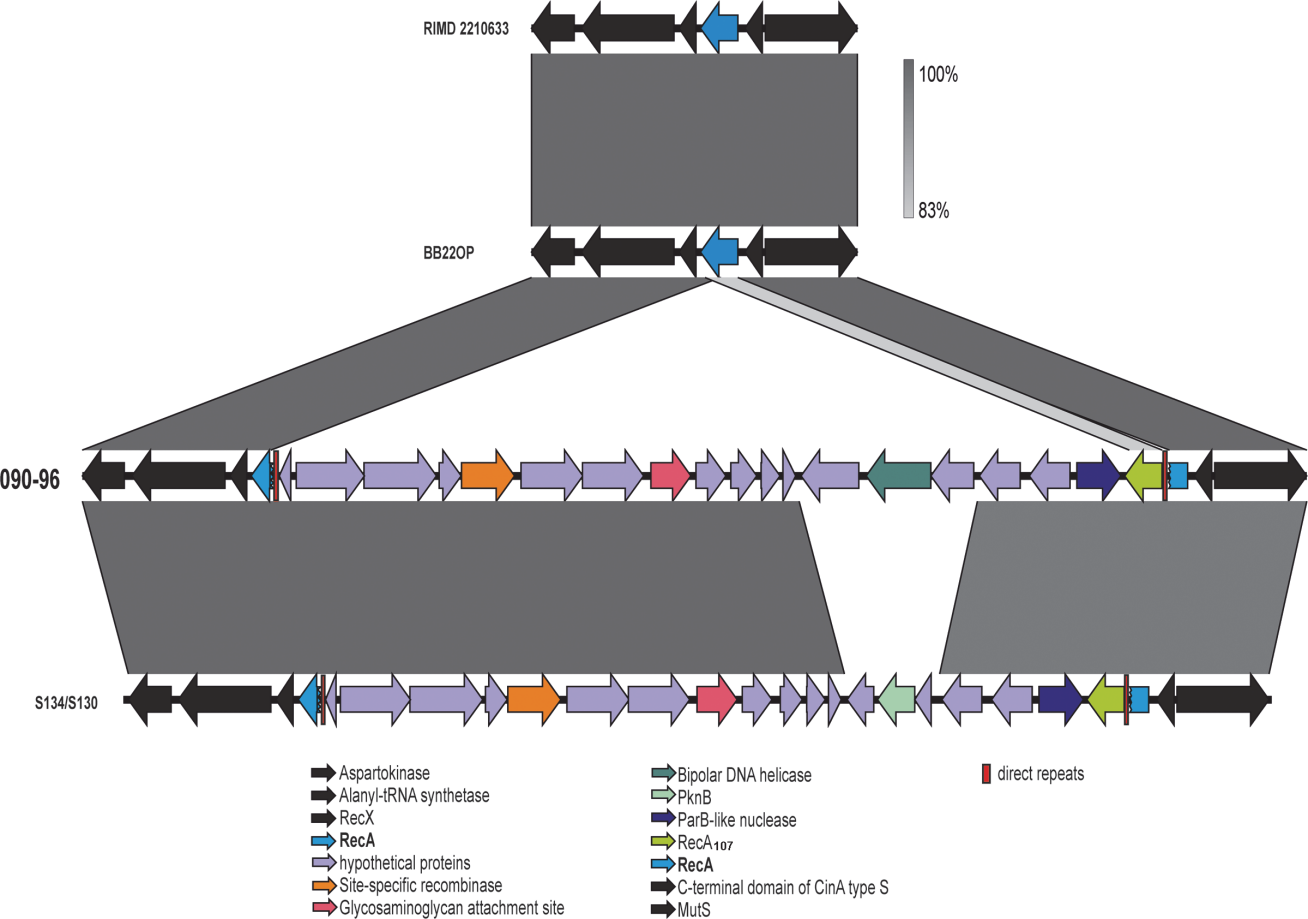
Schematic representation of the *recA* gene region between four *V*. *parahaemolyticus* genomes. Comparison includes RIMD2210633 (prototypic “pandemic” strain O3:K6), BB22OP (closest relative ST88), strains 090–96 (Peruvian, fragmented *recA* gene), and S134 (Chinese strain containing a similar insert at *recA* gene). Predicted genes are depicted by horizontal arrows and named according the legend. Red bars represent inverted regions flanking the insertion. The percentage DNA sequence identity is indicated in grey bars and represents the BLASTn matches between sequences.

**Analysis of the *recA* region in strain 090–96**. Sequencing of the whole genome of strain 090–96 provided the opportunity to examine the complete region flanking the *recA* gene. As shown in Fig. 2, the reported problems associated with the amplification of the *recA* gene of this strain was not due to the presence of a hybrid *recA*, as initially presumed, but instead was due to the insertion of a large element of ~30kb which disrupted the original *rec*A gene at nucleotide position 491 (Table 3). This inserted fragment had a G+C content of 38.8%, substantially lower than the average 45.3% G+C content for the *V*. *parahaemolyticus* genome. This fragment (30 kb) was probably acquired by HGT through an insertion event apparently using the *recA* gene as template or hotspot. The reconstruction of the original *recA* gene by the two *recA* fragments at either end of the insert overlapped by 6 bp (TCTCCA). Of the three annotation pipelines used, only RAST (http://rast.nmpdr.org/) was able to provide putative annotation of the genes within the inserted region (Table 3). RAST identified 19 ORFs in this region. This region included a ParB-like nuclease, a bipolar DNA helicase, a glycosaminoglycan attachment site, and a site-specific recombinase. Most of the genes in this region coded for currently unknown or hypothetical proteins. In addition to these genes, the inserted element also contained a complete novel *recA* gene that differed from the indigenous *V*. *parahaemolyticus recA* gene (recA3) by 24% and 23% of its nucleotide and inferred amino acid sequence, respectively. BLAST analysis was not able to identify any sequences highly similar to the inserted *recA* gene; the closest match was to a *recA* gene (90% identity at DNA sequence level) carried in a distinct mobile element (*recA* mobile element—RME) inserted in the indigenous *recA* gene of *V*. *cholerae* strain S24 [30]. The sequence of the *recA* variant carried in RME in *V*. *choleare* S24, group with other *recA* found in other members of the *Vibrio* genus within the *Vibrionaceae* family but distant from *V*. *choleare* or *V*. *parahaemolyticus* [30].

**Table 3:** Annotation of the *recA* region from the genome of strain 090–96 by RAST server [22].

Locus tag^a^	start	stop	Orientation	Putative protein identification
26	75090	72530	-	DNA mismatch repair protein MutS
25	75175	75657	+	CinA typeS
24	75858	76382	+	Truncated RecA protein
23	76558	77607	+	RecA protein
22	78935	77703	-	ParB-like nuclease
21	79126	80258	+	hypothetical protein
20	80786	81646	+	hypothetical protein
19	81808	83001	+	FIG036446: hypothetical protein
18	83002	84801	+	Bipolar DNA helicase
17	84994	86613	+	hypothetical protein
16	86880	86767	-	hypothetical protein
15	87684	87205	-	hypothetical protein
14	88575	87853	-	hypothetical protein
13	89542	88712	-	hypothetical protein
12	90799	89684	-	Glycosaminoglycan attachment site
11	92710	91005	-	hypothetical protein
10	94424	92694	-	hypothetical protein
9	94759	94598	-	hypothetical protein
8	96075	94768	-	Site-specific recombinase, phage integrase family
7	96817	96086	-	hypothetical protein
6	98795	96771	-	hypothetical protein
5	100734	98782	-	hypothetical protein
4	101400	101939	+	Truncated RecA protein
3	102063	102530	+	Regulatory protein RecX
2	102673	105255	+	Alanyl-tRNA synthetase (EC 6.1.1.7)
1	105458	106644	+	Aspartokinase (EC 2.7.2.4)

This region was identified in contig0035 (JFFP01000036) of the assembled shotgun genome. In bold fonts are the inserted genes probably by HGT. In green fonts are the two *recA* fragments resultants from the split of the original *recA*3.

^a^Order of the genes shown on Fig. 2.

Rapa et al. [30] described a similar genomic island insertion event into the *recA* gene of *V*. *cholerae* strain S24. However both regions differed greatly in organization, gene number and content. The RME from *V*. *cholerae* S24 contained a 9 bp inverted repeat at both ends while in the RME of *V*. *parahaemolyticus* 090–96 these repeats are of 6 bp. Furthermore, the RME from *V*. *cholerae* strain S24 (VcRME) contained 23 ORFs, compared to the 19 ORFs observed in the RME of the *V*. *parahaemolyticus* 090–96 strain examined in this study (VpRME). Most ORFs were annotated as “hypothetical” in VpRME while only 7 of the ORFs in the VcRME were annotated as hypothetical proteins [30]. In addition, there is an insertion sequence similar to ISVuv4containing transposase genes in the VcRME, in VpRME no insertion sequences elements were found (https://www-is.biotoul.fr), indicating that an unknown or undescribed mobile element could be present in the VpRME. Strikingly, the insertion occurred at a similar nucleotide position on the recA gene, 491 for recA_090–96_ and 494 for recA_S24_.

**Fig 3:**
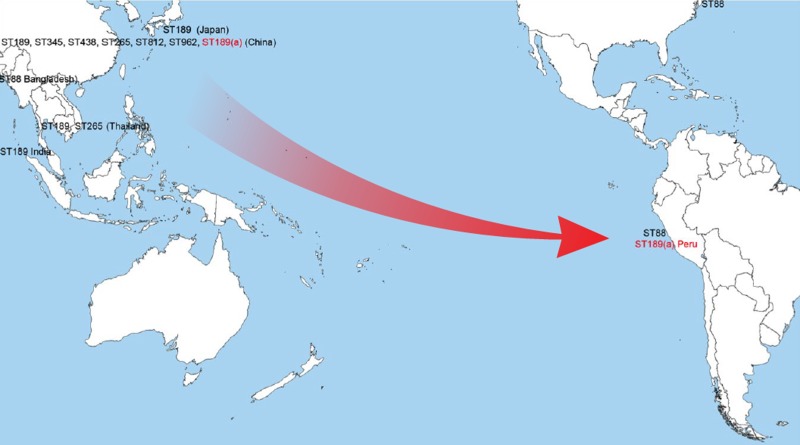
Sequence types (STs) identified at both side of the Pacific Ocean and route for the movement of waters associated with El Niño event (red arrow). STs in red indicate those genetic variants detected both in Asia and Peru (ST189a), including the ST189 for the strain 090–96 with the *recA* insertion.

The inserted *recA* variant (listed as recA107 in the *V*. *parahaemolyticu*s MLST website) has been previously identified in other *V*. *parahaemolyticu*s strains, all of which were isolated from Asian sources (Table 4 and Figure A in S1 File). The fact that a longer PCR fragment was observed when amplifying the *recA* gene (1400 bp, instead of 720 bp) was due to the fact that the forward *recA* primer failed to bind to its target region on the recA107 allele. However, it was able to anneal perfectly to the disrupted recA3 allele, together with the reverse primer annealing into the proximal recA107 produced a longer PCR product. A BLAST query of the inserted region against the whole genome shotgun contigs (wgs) available at GenBank identified two *V*. *parahaemolyticus* genomes that contained a highly similar insert that split the *recA* gene in the same manner observed in the 090–96 strain (Fig. 2). These two *V*. *parahaemolyticus* strains, S130 and S134, were isolated in Hong Kong from clinical samples in 2005 and 2003, respectively. Conversely, strains S130 and S134 differ in all the alleles from strain 090–96 and belonged to ST527. Finding insertion regions in two unrelated *V*. *parahaemolyticus* strains provides strong evidence that horizontal gene transfer (HGT) is responsible for the acquisition of this insert, and also helps explain the mobile nature of the insertion. Most likely, the insertion event used the *recA* gene as template or hotspot. The allele resultant from the reconstruction of the original *recA* gene for 090–96 strain was recA3 and combined with the rest of allelic profiles identified the ST189 as the precursor of the 090–96 and the other Peruvian strains with the same *recA* insertion (Table 2). Significantly, ST189 had never been identified in Peru and all reported *V*. *parahaemolyticus* strains currently belonging to ST189 have been isolated from East and South Asia, such as China, Thailand, India, and Japan (Fig. 3 and Table 2).

**Table 4:** *V*. *parahaemolyticus* strains with *recA* allele 107 available at the MLST *V*. *parahaemolyticus* database.

Strain	Year	Country	Source	dnaE	gyrB	recA	dtdS	pntA	pyrC	tnaA	ST
VP190	1990	Thailand	C	11	48	107	48	26	48	26	265
VP10339	2010	China	C	43	41	107	42	37	40	33	527
VP07001	2007	China	E	11	48	107	48	26	48	26	265
2011 37–11	2011	China	C	244	41	107	42	37	40	33	806
S134	2005	China	C	43	41	107	42	37	40	33	527
S130	2003	China	C	43	41	107	42	37	40	33	527

C- Clinical. E- Environmental.

Given the available information, we propose that the insertion event occurred somewhere in Asia in a common ancestor belonging to the ST189 lineage, with such organisms spreading among those regions before finally arriving near Peru around 1996, when the first strain with the insertion and allelic profile for ST189 was detected [16]. The introduction of strains belonging to the pandemic clonal complex followed a similar pattern, arriving in Peru between 1996 and 1997 [18,31], one year after its emergence in Southeast Asia. The dissemination of those pandemic strains along the coast of Peru was associated with the arrival and southward displacement of El Niño water in 1997 [18], which moved warm oceanic waters from Asia to Peru. The existence of this temporal, transoceanic connection between both sides of the Pacific Ocean may represent a recurrent route for the introduction of new pathogenic variants of *Vibrio* in the Peruvian coasts with the corresponding impact in the local populations and epidemiology of *Vibri*o diseases in the region [10,16] (Fig. 3). Furthermore, the existence of 8 additional strains with identical characteristics to strain 090–96 isolated in Peru associated with infections all along the coast of Peru over a period of 10 years provides additional evidence of the expansion of this genetic variant and its success in the Peruvian coastal waters, where it has become endemic.

**Fig 4:**
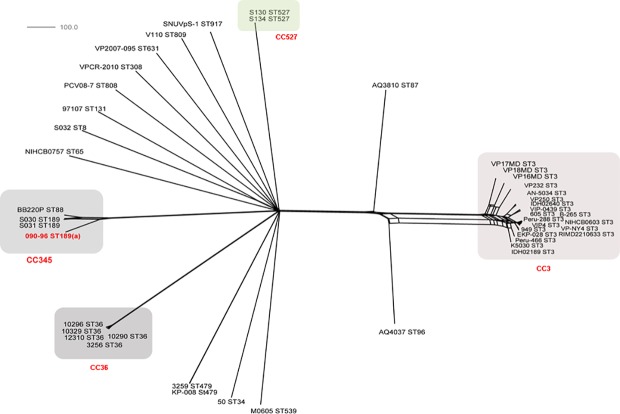
NeighborNet graph generated using the BIGSdb Genome Comparator tool implemented within the PubMLST website [34,35] with 2005 variable loci identified after using as a reference (ref) RIMD2210633 chromosome I (3079 genes) of available *V*. *parahaemolyticus* genomes at Genbank (Table A in S1 File for the list of the strains) and strain 090–96 (Peru 1996), showing that this strain is closed related to BB22OP (ST88) and the two other ST189 strains and far related to other strains containing fragmented *recA* genes with similar insertion (S130 and S134, both ST527). This clearly suggests acquisition of this region by HGT. A similar graph was obtained with chromosome II of the same strain as reference (data not shown). Distances between taxa are calculated as the number of loci with different allele sequences.

**Phylogenetic analysis of *V*. *parahaemolyticu*s genomes**. In order to assess the probable evolutionary changes associated with the acquisition of this fragment by HGT, all the available genomes of *V*. *parahaemolyticus* at GenBank and at the *V*. *parahaemolyticus* MLST database were analyzed using a wgMLST approach to infer the *V*. *parahaemolyticus* phylogeny from a broader context (Table A in S1 File). A matrix with 2004 variable loci from chromosome I was used to reconstruct the *V*. *parahaemolyticus* phylogeny for those strains. Confirming previous observations from MLST studies, the NeighborNet tree showed that *V*. *parahaemolyticus* is highly diverse and recombination events have occurred frequently on a whole genome scale, evidenced by the multiple splits present on the tree (Fig. 4) [11,14,17,29].

Based on these analyses, strains S130 and 134 were indistinguishable from each other and were phylogenetically unrelated to strain 090–96. They belonged to different *V*. *parahaemolyticus* clades, with 090–96 belonging to CC345 and S130/134 to CC527. This result supports the hypothesis that the 30 kb-insert in the *recA* gene was acquired through different HGT events in strain 090–96 and S130/S134 and this is probably an extended phenomenon among *V*. *parahaemolyticus* strains and possibly in other *Vibrio* species. As expected, strain 090–96 clustered by wgMLST with strains BB22OP (ST88), and with strains S030 (ST189) and S031 (ST189), supporting the results obtained with MLST that identified the ST189 as precursors of the strain 090–96. ST88 and ST189 differed in the *recA* allele, with 090–96, S030, and S031, having recA3 and BB22OP bearing the recA43. These two *recA* alleles differed by 27 nucleotides and according to empiric estimation these differences probably arose by recombination [11].

## Conclusions

The application of genome analyses to historical collections of pathogens is providing a novel dimension to epidemiological and outbreak investigations. The use of whole genome sequencing (WGS) in this study has revealed unexpected results in relation to the structure and genetic plasticity of the *recA* gene. This housekeeping gene is subject to horizontal gene transfer and its consideration as part of the typing schemes and genetic analysis with phylogenetic purposes needs to be revised, particularly in studies where this gene has been employed as unique source of genetic information to infer the phylogenic relationship and evolution of species belonging to the genus *Vibrio* [32],[33].

More significantly, this study has found that the initially deleterious disruption of a basic gene essential for the survival of the organism can be remediated through the insertion of a homologous gene from a related species. Perhaps this type of disruption is frequent in the environment; however, only in those cases which disruption is followed by a successful complementation, organisms would be able to survive and become detectable. The benefit that the inserted region and novel genes confer on *V*. *parahaemolyticus* strains in terms of ecological fitness, survival or virulence have not yet been elucidated. However, in a recent study, Rapa et al [30] suggested that this new *recA* could provide a protective effect against DNA damage induced by UV irradiation, contributing to an enhanced survival in the environment. Epidemiological data have shown that this novel variant has completely replaced the endemic O4:K8 clone in Peru since 1996 and it has been detected consistently all along the country afterwards. According to different models and data analysis, it is very likely that El Niño conditions will be developing during the boreal summer of this year (2014) and persisting into the autumn. The arrival of warm tropical waters to Peru moved by El Niño may contribute to the new introduction of new alien populations of pathogenic *Vibrio* in South America, but also provides a unique opportunity to evaluate the biological impact and public health implications of this event on Peru.

Finally, these results provide additional evidence supporting the existence of long distant dispersion of pathogenic *V*. *parahaemolyticus*, with the corresponding implications on the epidemiology of this disease at global scale. Since only few populations seem to be competent for long range spreading, comparative genomics and transcriptomics should enable the identification of biological attributes contributing to increased fitness for long-distance dispersals. The understanding of the gene and mechanisms controlling this basic process, combined with those associated with virulence, will be critical for the identification of those populations with a pandemic potential.

This work highlights the important role that genome sequencing and analysis will be playing for the characterization of clinical pathogenic bacteria and tracking the routes of dispersion of organisms and diseases. Recent outbreaks and associated investigation have shown that transoceanic spreading events can be more common than expected [15] and WGS data will play a critical role for verifying the geographical transoceanic spread of highly related strains and for delineating the routes and mechanisms of dispersion.

## Supporting Information

S1 File Fig. A. Minimum evolution tree of *recA* sequences in *V*. *parahaemolyticus* MLST website showing *recA* alleles that appear to be have been acquired by HGT because they matched *recA* genes from other isolates from the genus *Vibrio*. Sequences of recA107 (present in the VpRME) and recA3 (indigenous recA090-96) are in gray fonts in the phylogenetic trees. All sequences except for recAS24 were derived from V. parahaemolyticus; recAS24 was reported in a genomic island in V. cholerae strainS24. Values within parentheses represent the percent identity of each sequence to that of recA3 from V. parahaemolyticus. The inset tree is a sub-tree representing the distance between the wild-type (recA3) and HGT recA gene (recA107) in strain090-96. The scale bars represents 0.02 nucleotide substitutions per position. **Fig. B. *V*. *parahaemolyticus* “population snapshot” of all profiles available at the Vp MLST database obtained using eBURST v3**. One hundred twenty nine groups were defined using stringent criteria (6/7 shared alleles). CC345 can be seeing at the bottom. STs that are SLV of each other are shown connected by lines (black). DLV STs are shown as connected by gray lines. CC—Clonal Complex. **Table A. *V*. *parahaemolyticus* genomes used in the whole genome (wgMLST) comparator analysis**.
(DOCX)
