# Supplementary material for: Transoceanic Spreading of Pathogenic Strains of Vibrio parahaemolyticus with Distinctive Genetic Signatures in the recA Gene
Source: PLoS One. 2015 Feb 13;10(2):e0117485. doi: 10.1371/journal.pone.0117485 (PMC4334540; doi:10.1371/journal.pone.0117485)
Supplement: S1 File — Sequences of recA107 (present in the VpRME) and recA3 (indigenous recA090-96) are in gray fonts in the phylogenetic trees. All sequences except for recAS24 were derived from V. parahaemolyticus; recAS24 was reported in a genomic island in V. cholerae strainS24. Values within parentheses represent the percent identity of each sequence to that of recA3 from V. parahaemolyticus. The inset tree is a sub-tree representing the distance between the wild-type (recA3) and HGT recA gene (recA107) in strain090-96. The scale bars represents 0.02 nucleotide substitutions per position. Fig. B. V. parahaemolyticus “population snapshot” of all profiles available at the Vp MLST database obtained using eBURST v3. One hundred twenty nine groups were defined using stringent criteria (6/7 shared alleles). CC345 can be seeing at the bottom. STs that are SLV of each other are shown connected by lines (black). DLV STs are shown as connected by gray lines. CC—Clonal Complex. Table A. V. parahaemolyticus genomes used in the whole genome (wgMLST) comparator analysis. (DOCX) [file pone.0117485.s001.docx]

**Supplementary Tables AND Supplementary Figures**



**Supplementary Figure** **A**. Minimum evolution tree of *recA* sequences in *V. parahaemolyticus* MLST website showing *recA* alleles that appear to be have been acquired by HGT because they matched *recA* genes from other isolates from the genus *Vibrio*. Sequences of recA107 (present in the VpRME) and recA3 (indigenous recA_090-96_) are in gray fonts in the phylogenetic trees. All sequences except for recAS24 were derived from *V. parahaemolyticus*; recAS24 was reported in a genomic island in *V. cholerae* strainS24. Values within parentheses represent the percent identity of each sequence to that of recA3 from *V. parahaemolyticus*. The inset tree is a sub-tree representing the distance between the wild-type (recA3) and HGT *recA* gene (recA107) in strain090-96. The scale bars represents 0.02 nucleotide substitutions per position.





**Supplementary Figure** **B**. *V. parahaemolyticus* “population snapshot” of all profiles available at the Vp MLST database obtained using eBURST v3. One hundred twenty nine groups were defined using stringent criteria (6/7 shared alleles). CC345 can be seeing at the bottom. STs that are SLV of each other are shown connected by lines (black). DLV STs are shown as connected by gray lines. CC – Clonal Complex.

**

**

**Supplementary Table A** – *V. parahaemolyticus* genomes used in the whole genome (wgMLST) comparator analysis**.**

| **Strain**  **denomination** | **NCBI**  **accession No.** |  | **Sequencing**  **status** | **Size (Mb)** | **GC**  **Content (%)** | **ST** | **Country/**  **Region** | **Year** | **Source** | ***tdh*** | ***trh*** |
| --- | --- | --- | --- | --- | --- | --- | --- | --- | --- | --- | --- |
| 10329 | NZ_AFBW01000000 |  | SC | 5.09 | 45.3 | 36 | USA/WA | 1998 | C | + | + |
| RIMD 2210633 | BA000032.2, BA000031.2 (NC_004603, NC_004605) |  | complete | 5.17 | 45.4 | 3 | Japan/OS | 1996 | C | + | - |
| BB22OP | CP003973.1, CP003972.1 |  | complete | 5.1 | 45.3 | 88 | Bangladesh | ~1980 | E |  |  |
| AN-5034 | ACFO00000000 |  | SC | 5.2 | 45.3 | 3 | Bangladesh | 1998 | ? | + | - |
| AQ3810 | AAWQ00000000 |  | SC | 5.78 | 45.6 | 87 | Singapore | 1983 | C |  |  |
| AQ4037 | ACFN00000000 |  | SC | 4.94 | 45.4 | 96 | Maldives | 1985 | ? |  |  |
| K5030 | ACKB00000000 |  | SC | 5.03 | 45.3 | 3 | ? | 2005 | ? | + | - |
| PCV08-7 | AOCL00000000 |  | SC | 5.18 | 45.3 | 808 | Malaysia | 2008 | E |  |  |
| Peru-466 | ACFM00000000 |  | SC | 5.04 | 45.4 | 3 | Peru | 1996 | ? |  |  |
| SNUVpS-1 | AMRZ00000000 |  | SC | 5.24 | 45.2 | 917 | Korea | 2009 | E |  |  |
| v110 | AQPJ00000000 |  | SC | 5.51 | 45.2 | 809 | China | 2010 | E |  |  |
| 3259 | AVOL01000000 |  | SC | 5.36 | 45.2 | 479 | USA | 2007 | C |  |  |
| 949 | AVPV01000000 |  | SC | 5.18 | 45.4 | 3 | USA | 2006 | C |  |  |
| NIHCB0603 | AVOM00000000 |  | SC | 5.23 | 45.4 | 3 | Bangladesh | 2006 | C |  |  |
| NIHCB0757 | AVPX01000000 |  | SC | 5.26 | 45.3 | 65 | Bangladesh | 2006 | C |  |  |
| VP-NY4 | AVON01000000 |  | SC | 5.25 | 45.4 | 3 | India | 1997 | C |  |  |
| VP2007-095 | NZ_AVOI01000000 |  | SC | 5.18 | 45.3 | 631 | USA | 2007 | C |  |  |
| VP232 | NZ_AVOJ01000000 |  | SC | 5.12 | 45.4 | 3 | India | 1998 | C |  |  |
| VP250 | NZ_AVOK01000000 |  | SC | 5.2 | 45.4 | 3 | India | 1998 | C |  |  |
| VPCR-2010 | NZ_AVPW01000000 |  | SC | 6.08 | 44.1 | 308 | USA | 2010 | E |  |  |
| Peru-288 | JAII01000000 |  | SC | 5.19 | 45.3 | 3 | Peru | 2001 | C |  |  |
| VIP4-0395 | AXNJ00000000 |  | SC | 5.12 | 45.3 | 3 | Hong Kong | 2007 | C |  |  |
| VIP4-0439 | AXNK00000000 |  | SC | 5.14 | 45.3 | 3 | Hong Kong | 2008 | C |  |  |
| 10296 | AYSP01000000 |  | SC | 5.13 | 45.3 | 36 | WA | 1997 | C |  |  |
| 12310 | AYXP00000000 |  | SC | 5.15 | 45.3 | 36 | WA | 2006 | C |  |  |
| 3256 | AZGS00000000 |  | SC | 5.1 | 45.3 | 36 | USA | 2007 | C |  |  |
| 50 | AZGT00000000 |  | SC | 5.07 | 45.2 | 34 | USA | 2006 | C |  |  |
| 605 | AZGU00000000 |  | SC | 5.16 | 45.3 | 3 | USA | 2006 | E |  |  |
| 970107 | AZIQ00000000 |  | SC | 5.12 | 45.3 | 131 | USA | 1997 | E |  |  |
| B-265 | AZKN00000000 |  | SC | 5.2 | 45.3 | 3 | Mozambique | 2004 | C |  |  |
| EKP-008 | AZMN00000000 |  | SC | 5.32 | 45.2 | 479 | Bangladesh | 2007 | E |  |  |
| EKP-028 | JACH00000000 |  | SC | 5.21 | 45.3 | 3 | Bangladesh | 2008 | E |  |  |
| IDH02189 | JAHD00000000 |  | SC | 5.11 | 45.3 | 3 | India | 2009 | C |  |  |
| IDH02640 | JAIH00000000 |  | SC | 5.23 | 45.1 | 3 | India | 2009 | C |  |  |
| 10290 | AVOH00000000 |  | SC | 5.18 | 45.4 | 36 | USA/WA | 1997 | C |  |  |
| Vp16MD | Vp MLST db |  | SC | 5.10 | 45.3 | 3 | USA/MD | 2012 | C |  |  |
| Vp17MD | Vp MLST db |  | SC | 5.10 | 45.3 | 3 | USA/MD | 2012 | C |  |  |
| Vp18MD | Vp MLST db |  | SC | 5.10 | 45.3 | 3 | USA/MD | 2012 | C |  |  |
| S032 | AWMD00000000 |  | SC | 5.12 | 45.2 | unk |  |  |  |  |  |
| S130 | AWIW00000000 |  | SC | 5.19 | 45.3 | 527 | Hong Kong | 2005 | C |  |  |
| S134 | AWIS00000000 |  | SC | 5.19 | 45.3 | 527 | Hong Kong | 2003 | C |  |  |
| S030 | AWMF01000000 |  | SC | 5.09 | 45.2 | 189 | India | 1999 | C |  |  |
| M0605 | JALL00000000 |  | SC | 5.43 | 45.2 | 539 | Mexico | 2013 | E |  |  |
| S031 | AWME00000000 |  | SC | 4.99 | 45.3 | 189 | Japan | 1984 | C |  |  |

SC- Scaffolds or contigs.

ST- Sequence type.

Vp MLST db- *V. parahaemolyticus* MLST database (http://pubmlst.org/vparahaemolyticus/)
